# Cutaneous Bacillary Angiomatosis: A Rare and Forgotten Infection in Immunocompromised Patient

**DOI:** 10.1055/s-0045-1812318

**Published:** 2025-10-28

**Authors:** Yamen Homsi, Emily Milam, Nour Dayoub

**Affiliations:** 1 Department of Rheumatology, NYU Grossman School of Medicine, New York, New York, United States; 2 Department of Dermatology, NYU Grossman School of Medicine, New York, New York, United States; 3 Lang Research Center, NewYork-Presbyterian Queens Hospital, Queens, New York, United States

**Keywords:** bacillary angiomatosis, angioproliferative lesions, *Bartonella* infection, immunocompromised patients, renal transplant recipients

## Abstract

This case describes a 50-year-old kidney transplant recipient with subacute development of erythematous-to-violaceous skin lesions on the face, trunk, and extremities, accompanied by malaise, myalgia, and arthralgia. Histopathologic analysis of skin biopsies revealed characteristic vascular proliferation consistent with bacillary angiomatosis (BA), a rare angioproliferative disease caused by *Bartonella* *henselae* or *Bartonella* *quintana* infection, primarily affecting immunocompromised individuals. The patient was treated successfully with oral doxycycline, resulting in the resolution of symptoms and lesions. BA is typically transmitted via cats and presents variably, including cutaneous angioproliferative lesions, hepatic or splenic involvement, and endocarditis. Diagnosis relies on histopathology with specialized staining and molecular testing, as culture and serologies are often insufficient. Treatment typically involves prolonged antibiotic therapy, emphasizing the importance of early recognition in immunosuppressed patients, including solid organ transplant recipients, to prevent complications.

## Introduction

Bacillary angiomatosis (BA) is a rare angioproliferative disease of immunocompromised patients caused by the gram-negative bacilli *Bartonella henselae* and *Bartonella quintana* . BA causes angiomatous lesions that mainly affect the skin but can involve internal organs such as the spleen and liver. Herein, we report a case of cutaneous BA in a kidney transplant recipient who presented with the subacute development of skin lesions and associated malaise, myalgia, and arthralgia.

## Case

A 50-year-old Turkish man presented to the clinic for evaluation of a 1- to 2-month history of enlarging skin lesions on his face, trunk, and extremities. He also noted concurrent malaise, myalgia, and arthralgia, but denied fevers, chills, headache, chest pain, shortness of breath, or abdominal pain. His medical history was notable for granulomatosis with polyangiitis with renal involvement that failed treatment with pulse steroids and rituximab. He briefly required hemodialysis and ultimately underwent kidney transplantation. At the time of presentation, he was on tacrolimus, mycophenolate mofetil, and prednisone. He denied any recent travel, new sexual partners, or illicit drug use. He worked as an Uber driver, and his only exposure to animals was when passengers brought their cat or dog into his car.

**Fig. 1:**
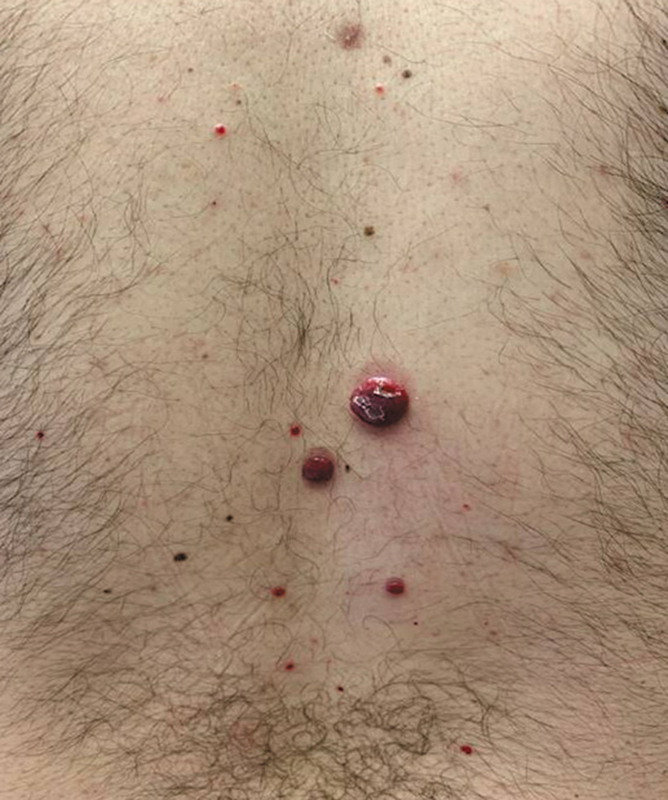
Multiple raised, red-to-purple nodules of varying sizes scattered on the patient's back.

**Fig. 2:**
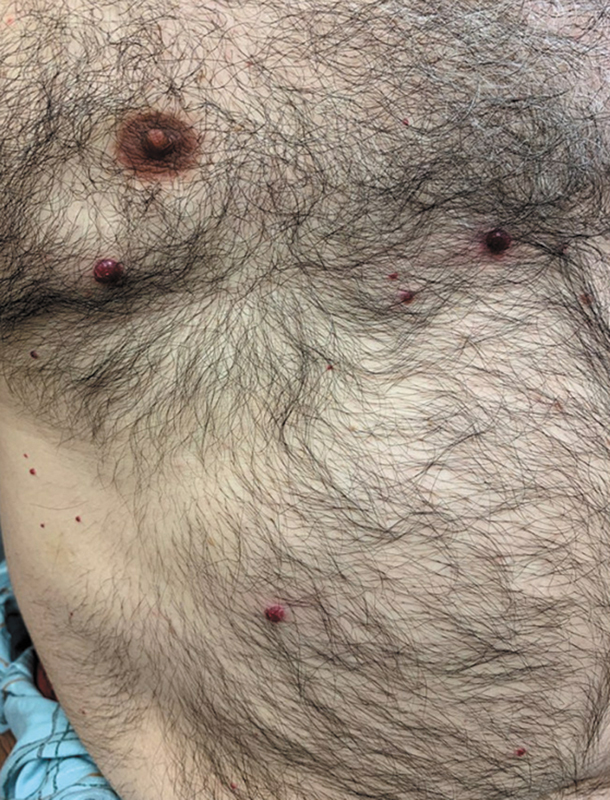
A photo of similar lesions noted on the chest and abdomen.

Physical examination was notable for numerous brightly erythematous-to-violaceous, firm papules and ulcerated, exophytic nodules of varying sizes on his face, trunk, and extremities ( Figs. 1 and 2 ). On the dorsal aspect of his left forearm was a palpable, mildly tender subcutaneous nodule. His oral mucosa was unremarkable. There was no cervical, submandibular, axillary, or inguinal lymphadenopathy. Lung, heart, abdominal, and joint examinations were within normal.

A complete blood count revealed a mild neutrophilia (77%, normal range 34–68%) and decreased lymphocytes (10%, normal range 22–53%), but was otherwise within acceptable ranges. A complete metabolic panel was normal. Tests for hepatitis A, hepatitis B, hepatitis C, and human immunodeficiency virus (HIV) were negative. A QuantiFERON-TB Gold test was negative.

**Fig. 3:**
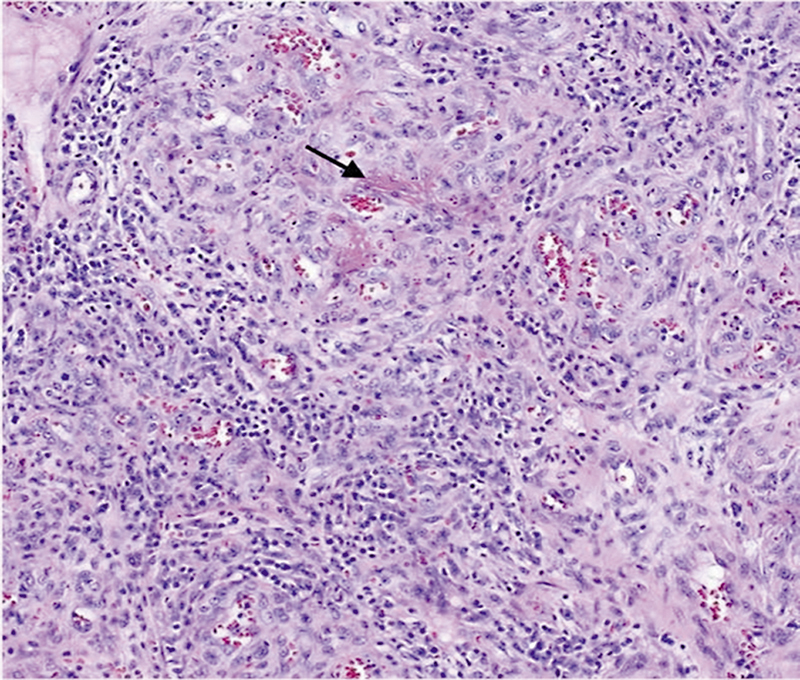
Histopathological features of bacillary angiomatosis, shave biopsy sample; lobular proliferation of blood vessels with deposits of amorphous eosinophilic material in the stroma.

Shave skin biopsies were obtained from lesions on the nose and back. Histopathologic examination revealed a lobular proliferation of blood vessels with deposits of amorphous eosinophilic material in the stroma, which were highlighted by a Steiner stain, overall consistent with a diagnosis of BA ( Fig. 3 ). Tissue cultures and acid-fast bacilli staining were negative. Serologic testing for *Bartonella* immunoglobulin G and immunoglobulin M antibodies was indeterminate.

The patient started oral doxycycline 100 mg twice daily for 3 months, which quickly diminished the size of existing lesions and ultimately resulted in complete resolution of all lesions and symptoms. Immunosuppressive therapy was maintained without dose adjustment or discontinuation of any agent.

## Discussion

We report a case of a middle-aged kidney transplant recipient who presented with the subacute development of angioproliferative lesions on his face, trunk, and extremities, with associated malaise, myalgias, and arthralgias. While a *Bartonella* species could not be cultured or confirmed by serologic testing, skin biopsy revealed a characteristic lobular vascular proliferation and deposits of amorphous eosinophilic material highlighted by a Steiner stain, overall diagnostic of cutaneous BA.

### *Bartonella* Species

*Bartonella henselae* is the most common culprit of cutaneous BA. *Bartonella henselae* is primarily an infectious agent of cats, the primary reservoir, that is transmitted through the feces of the infected cat flea, *Ctenocephalides felis* . 1 2 Humans represent incidental hosts that become infected by direct inoculation of cat flea feces into the skin, typically via a cat scratch. Serologic studies indicate that infection of domestic cats is worldwide, with the highest prevalence in warm, humid climates. Other studies have shown that infection with *B. henselae* is possible through *Stomoxys* sp. (biting flies), rodent lice, 3 and *Ixodes* *ricinus* saliva. 4

*Bartonella henselae* infection can manifest in a variety of ways, including the classic self-limited regional lymphadenopathy (“cat scratch disease”), cutaneous angioproliferative lesions (“bacillary angiomatosis”), hepatic and splenic angioproliferative lesions (“bacillary peliosis”), blood culture–negative endocarditis, neuroretinitis, encephalopathy, osteomyelitis, and as a cause of fever of unknown origin in immunocompetent or immunocompromised patients. 1

### Cutaneous BA: Diagnosis

As seen in this patient, cutaneous BA lesions are often multiple (sometimes exceeding 1,000 lesions) and widely distributed on the skin with varying sizes and morphologies. Lesions may involve subcutaneous tissue, bones, mucosa, and/or internal organs, and larger cutaneous lesions can become ulcerated, friable, and bleed significantly. As such, cutaneous lesions may resemble pyogenic granulomas, cherry angiomas, Kaposi sarcoma, fungal infections, and nontuberculous mycobacterial infections. 2

Histopathology specimens stained with hematoxylin and eosin typically reveal a characteristic lobular vascular proliferation, with rounded vessels lined by plump endothelial cells and a predominantly neutrophilic infiltrate. A Warthin–Starry or a Steiner stain aids in highlighting *Bartonella* bacilli within the tissue. *Bartonella* is notoriously difficult to culture, and thus, a negative culture should not discount the diagnosis. 5 *Bartonella* serologies are also inconsistently helpful, as antibody production of those infected can be variable. Polymerase chain reaction is available and a more dependable testing method.

### BA in Immunocompromised Hosts

Cutaneous BA and other forms of pathologic, widespread vasoproliferation of *B. henselae* organisms are more common among immunocompromised hosts. 6 7 Historically, this presentation was principally seen in patients with HIV/AIDS; however, other immunosuppressed individuals, such as solid organ transplant (SOT) recipients, represent an emerging, susceptible patient group, 8 9 as seen in our patient.

In a review of 29 SOT recipients who developed *B. henselae* infection—two-thirds of whom had undergone kidney transplant—the majority (72%) demonstrated disseminated disease, while 28% had typical cat-scratch disease. 9 Prominent clinical features included fever (93% of patients), lymphadenopathy (41%), and skin lesions (24%). The mean time between organ transplantation and presentation of *Bartonella* infection among those with cat-scratch disease was 5.6 ± 5.3 years, and among those with disseminated infection was 2.7 ± 2.4 years. Notably, most patients owned a cat or had exposure to cats. One patient had good evidence of donor-derived *Bartonella* infection.

### BA in Immunocompetent Hosts

Although rare, BA can occur in immunocompetent hosts, presenting as localized single or a few cutaneous tender, erythematous, papulonodular eruptions, typically less extensive and severe compared with those in immunocompromised patients. 10 11

Systemic involvement in immunocompetent hosts is uncommon but has been reported in some cases; symptoms were reported to be abdominal pain, splenomegaly, hepatomegaly, adenopathy, and chest pain. 10

## Conclusion

This case report highlights the importance of considering BA in the differential diagnosis of angioproliferative lesions in immunocompromised patients, even in the absence of clear exposure to typical risk factors, such as cat scratches or bites. There may be a potential risk of donor-derived infection, as other reports have shown. 12 The diagnostic challenge posed by this case is evident by the seronegative *Bartonella* testing, which emphasizes the pivotal role of histopathologic examination and tissue staining, such as Steiner or Warthin–Starry stains, in confirming the diagnosis. Additionally, this report points out the need for heightened awareness of BA among SOT recipients, who represent a vulnerable and emerging patient population ( Table 1 ).

**Table 1:** Comparing our case with other studies in the past 5 years

Case	Our case	Brzewski et al 13	Morillas et al 14	Eid et al 15	Mehrmal et al 16
Age (y)	50	65	67	75	37
Sex	Male	Male	Male	Female	Male
Immunosuppressive regimen	Tacrolimus Mycophenolate mofetil Prednisone	Tacrolimus 6 mg/d Mycophenolate mofetil 2 g/d Prednisone 5 mg/d	Tacrolimus Mycophenolate mofetil Prednisone	Prednisone Mycophenolate mofetil Belatacept infusions/monthly	Tacrolimus Mycophenolate mofetil Prednisone
Exposure history	No direct exposure to cats	Not available	Cat exposure 8 years prior to kidney transplant	Brief cat exposure, no scratches or bites	Cat exposure confirmed
Presenting symptoms	1- to 2-month history of enlarging skin lesions on face, trunk, and extremities Malaise, myalgia, and arthralgia No fevers, chills, headache, chest pain, shortness of breath, or abdominal pain	High fever and numerous nodular skin lesions Sparing mucosa and genital areas	Fevers, night sweats, fatigue Poor appetite and weight loss 2 months later: Multiple, nontender, red-violaceous papules Progressed from localized to disseminated	3-month history of intermittent fever, chills, night sweats, vomiting, and a 12-kg weight loss No skin lesions were identified	2-week history of fevers, chills, anorexia, weight loss, abdominal pain, diarrhea New, asymptomatic lesion on the right neck
Test results	Serologic testing for *Bartonella* IgG and IgM antibodies were indeterminate	Increased WBC count Elevated values of acute phase reactants Negative blood cultures	16S rDNA primer on tissue sample was negative BH IgG and BQ IgG by indirect fluorescent antibody were indeterminate BH IgM and BQ IgM were negative	Blood and urine cultures were negative Serologic tests for *Bartonella* were also negative using an immunofluorescence assay test (PET) CT scan showing a hypermetabolic mass in the duodenopancreatic region, with multiple hepatic and splenic hypermetabolic lesions	Blood cultures and other infectious workup were negative Serologic testing for *B* . *henselae* was positive Skin lesion biopsy, Warthin–Starry stain revealed scattered coccobacilli DPAS, Gram, and acid-fast were negative for microorganisms
Diagnosis	Positive skin lesion biopsy	Positive skin lesion biopsy	Positive blood *Bartonella* qualitative PCR	Positive PCR of a celiac lymph node tissue biopsy	Skin lesion biopsy and positive serology
Treatment regimen	Oral doxycycline 100 mg twice daily for 3 months	Oral doxycycline 100 mg twice a day for 3 months	Mycofenolate mofetil dose was reduced Doxycycline 100 mg twice a day Treated for 12 months total	IV gentamicin 3 mg/kg/d + oral doxycycline 100 mg twice daily for 2 weeks for initial suspicion of endocarditis Then oral doxycycline 100 mg twice daily for 3 months	Oral doxycycline 100 mg twice daily for 3 months
Outcome	Complete resolution	Complete resolution for 1 year without relapse	Complete resolution for 6 months without relapse	Relapse 3 months after Doxycycline cessation Mycophenolate mofetil was discontinued Erythromycin (1 g twice daily) + rifampin (600 mg twice daily) for 6 weeks Complete resolution for 1 year without relapse	Resolution of symptoms after 2 weeks of treatment initiation No long-term follow-up was documented

Abbreviations: BH, *Bartonella* *henselae* ; BQ, *Bartonella* *quintana* ; CT, computed tomography; DPAS, diastase-periodic acid–Schiff stain; IgG, immunoglobulin G; IgM, immunoglobulin M; PET, positron emission tomography; PCR, polymerase chain reaction; WBC, white blood cell.
